# Neglect of Older Adults Living with Dementia in Family Caregiving: A Dimensional Concept Analysis

**DOI:** 10.1177/00912174241272615

**Published:** 2024-08-12

**Authors:** Jessica A Hernandez Chilatra, Patricia A Patrician, Pariya L Fazeli, Carolyn EZ Pickering

**Affiliations:** 1 McWilliams School of Biomedical Informatics, 12340 The University of Texas Health Science, Houston, TX, USA; 2 Department of Research, Cizik School of Nursing, 12340 University of Texas Health Science Center at Houston, Houston, TX, USA; 3 School of Nursing, 9968 The University of Alabama at Birmingham, Birmingham, AL, USA

**Keywords:** Elder neglect, older population, gerontological research, dementia family, caregiving, nursing research

## Abstract

**Objective:**

To present a concept analysis of neglect, specifically examining its occurrence and implications in the context of family caregiving for older adults living with dementia.

**Methods:**

A literature search was conducted in Medline, CINAHL, Scopus, and Embase databases in February 2023. Inclusion criteria targeted articles focusing on neglect in dementia family caregiving, leading to the identification of 11 articles for thorough review. Employing Caron and Bowers’ dimensional analysis approach, the concept analysis aimed to elucidate neglect as a social construct shaped by diverse contexts, perspectives, and underlying assumptions.

**Results:**

Neglect in this context emerged as a multidimensional phenomenon, influenced by contextual elements such as activities of daily living and behavioral symptoms of dementia. It encompasses dimensions including “expectations of unmet needs”, “maladaptive behaviors”, and “feelings of guilt”, considering the perspectives of both caregivers and individuals living with dementia. Recognizing neglect as a dyadic phenomenon emphasizes the significance of interactions between caregivers and individuals living with dementia.

**Conclusion:**

A comprehensive understanding of neglect in dementia family caregiving is crucial for effective interventions and support systems. The dyadic perspective is vital for accurate assessment. Primary care physicians, mental health, nurses, and other health professionals play a key role in prevention and supporting family caregivers. Further research is needed to explore the dynamics of dementia caregiving settings strengthening prevention strategies against elder neglect.

## Introduction

The concept of neglect within the context of dementia family caregiving is a complex and significant issue that requires in-depth exploration and understanding. Elder abuse, encompassing neglect, represents a significant public health concern, especially affecting the rapidly growing population of individuals aged 60 and above.^1,2^ Common types of abuse of older adults include but are not limited to many forms of abuse (physical, sexual, psychological, and emotional abuse; financial and material abuse) and neglect.^3^

Elder abuse and neglect prevalence is higher among community-dwelling older adults with dementia, ranging from 50%–70%.^4-6^ In the United States, more than 6.5 million people live with dementia, with around eleven million informal caregivers, predominantly family and friends, offering unpaid care.^7-9^ These caregivers face unique challenges and burdens, increasing their risk of physical, emotional, and financial problems, potentially leading to the use of neglectful behaviors.^4,10,11^

Neglect, as a form of elder abuse, poses a particular challenge in defining and measuring its occurrence. The absence of a standardized tool for measuring neglect further complicates research efforts, as many studies depend on assessments that do not consider the caregiving context. Moreover, neglect in the context of dementia family caregiving must be examined from a dyadic perspective, considering the interactions between the caregiver and the person living with dementia.^12^

To address these challenges and develop a conceptual understanding of neglect within the dementia family caregiving context, this concept analysis adopts the dimensional analysis approach proposed by Caron and Bowers.^13^ This method is particularly advantageous as it views concepts not as static entities, but as dynamic social constructs. These constructs are shaped and influenced by different factors, including diverse contexts, varying perspectives of those involved (such as caregivers and older adults living with dementia), and prevailing societal assumptions about caregiving and dementia (such as beliefs about the roles and responsibilities of family caregivers). By employing this method, the purpose of this concept analysis is to describe elder neglect as a social concept, explore its multiple perspectives within the context of dementia family caregiving, differentiate the relationships between these perspectives, and identify underlying assumptions.

### Background

Almost half of caregivers in the US are providing support to individuals who are living with Alzheimer’s or other forms of dementia.^7^ These informal caregivers are mostly family members or friends,^8,9^ and their presence is key to maintaining the quality of life of their relative with dementia.^14^ Those diagnosed with dementia experience progressive disability and loss of independence, requiring higher levels of medical and supportive care over time,^1^ which in turn represents higher costs to the family and society.^15^

Caregivers of older adults living with dementia face unique hardships, often leading to physical, emotional, and financial challenges that are considerably greater than those faced by caregivers of individuals with other conditions.^7,16-18^ Research indicates that the stress associated with managing dementia symptoms can lead to adverse outcomes for both the patient and the caregiver, including increased risk of neglect.^4,10,11,19-21^

Currently, there are different definitions proposed for neglect as a form of elder abuse. The World Health Organization^22^ defines neglect as a single or repeated act, or lack of action, within a trust-based relationship, causing harm or distress to an older person. Moreover, The Centers for Disease Control and Prevention^23^ define elder neglect as the “failure to meet an older adult’s basic needs”.^23^ However, alternative definitions of neglect in the older adults’ context place the responsibility on caregivers. According to the National Institute on Aging,^24^ elder neglect focuses on the caregiver’s failure to respond to the older adult’s needs. When elder neglect occurs within a caregiving context it usually involves the failure to facilitate or provide basic needs such as nutrition, hygiene, or medical care.^25^ The National Council of Aging,^26^ establishes the definition of “passive neglect” as cases in which caregivers do not provide an older adult with the basic necessities of life.

Existing definitions of elder neglect often carry a punitive tone, portraying caregivers primarily in a negative light, as perpetrators. However, this oversimplified view overlooks the complex, dyadic nature of the caregiver-older adult living with dementia relationship, which can significantly influence neglectful outcomes. According to Dong,^27^ there is a limit to how much we can trust prevalence data based on the lack of consensus and different definitions of abuse and neglect, cultural backgrounds, measurement tools settings, and study populations.

Neglect, unlike other types of elder abuse, is inherently tied to the caregiving context. It’s a unique challenge in terms of definition and measurement, as it cannot be detached from the caregiving situation. Traditional research on physical and psychological abuse often employs tools like the Conflict Tactics Scale – Revised^28^ to measure interpersonal aggression. However, there is no equivalent tool for accurately measuring neglect. As a result, many studies resort to basic assessments, such as checking whether caregivers have omitted essential care tasks like medication reminders or meal preparation. It is crucial to understand that such ‘skipped care' is distinct from neglect, particularly among formal caregivers.^29^ This involves recognizing that while skipped care can be a manifestation of neglect, it is not synonymous with it. Moreover, what constitutes neglect among older adults can vary by culture and context.^30^

This concept analysis efforts to explore neglect, focusing specifically on the caregiver-person with dementia dyad, rather than treating it as a broad, generic phenomenon. This approach is critical for recognizing the multifaceted nature of caregiving relationships where neglectful behaviors might arise, not out of malicious intent, but as a response to complex environmental and relational dynamics. By dissecting and redefining neglect within this specific context, this analysis addresses a crucial gap in existing research. It aims to shift the narrative from a punitive perspective of caregivers towards a more empathetic understanding of the challenges they face. This nuanced understanding is vital not only for protecting older adults with dementia but also for informing the development of supportive strategies and interventions that can aid caregivers in managing their responsibilities without resorting to neglectful practices. The goal is to offer a comprehensive and contextualized conceptualization of neglect, as explored in health-related literature, thereby contributing a significant and much-needed perspective in the realm of dementia family caregiving. This study’s findings could guide the quality of future research, the development of context-sensitive measurement tools, policy-making, and clinical practice, ensuring better care and support for both individuals with dementia and their caregivers.

## Methods

### Aim

This study aimed to carry out an analysis of the concept of neglect within the context of dementia family caregiving. The specific questions proposed for analysis were: (a) What is the nature of ‘neglect’ within the context of dementia family caregiving; (b) What are the dimensions of ‘neglect’ within the context of dementia family caregiving? and (c) What are the perspectives reflected in each study?

### Data sources

**Figure 1.**
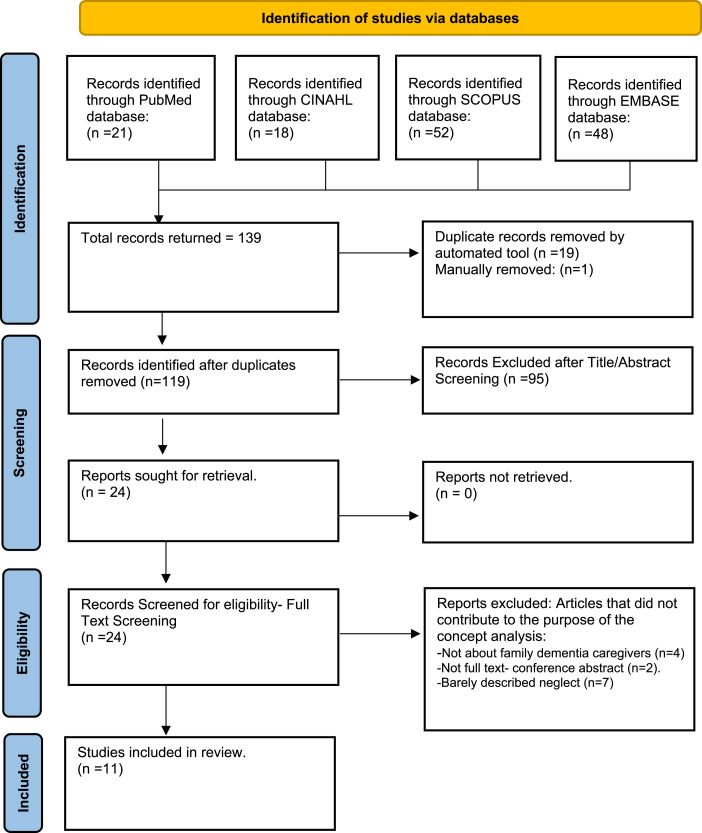
Literature search strategy to identify articles for the concept analysis. From: Page MJ, McKenzie JE, Bossuyt PM, Boutron I, Hoffmann TC, Mulrow CD, et al. The PRISMA 2020 statement: an updated guideline for reporting systematic reviews. BMJ 2021;372:n71. doi: 10.1136/bmj.n71. For more information, visit: https://www.prisma-statement.org/.

Figure 1 illustrates the literature search process. Three databases were used to search the literature: Medline, The Cumulative Index to Nursing and Allied Health Literature (CINAHL), and Scopus. The search was conducted in February 2023. A comprehensive search was conducted using a combination of 3 key terms: ‘Neglect,’ ‘Family/Informal Caregiver,' and ‘Dementia.' These terms were integrated into the following search query: The query included ‘Neglect' and ‘Elder neglect' in the title or abstract, combined with either ‘Family caregiver’ or ‘Informal caregiver' in the same fields. Additionally, ‘Dementia’ as a MeSH term or ‘Dementias' in the title or abstract were included. To ensure specificity, filters were applied to exclude results related to ‘Child Abuse,’ ‘Pediatrics,’ and ‘Adolescent,’ either as MeSH terms or in the title or abstract. Results were subsequently limited to “published within the previous 10 years”. This search resulted in 139 peer-reviewed journal articles for review. Abstracts, titles, and subject classifications were reviewed thoroughly. Inclusion criteria were articles addressing neglect or elder neglect of people living with dementia within the family caregiving context. Articles were excluded when they did not contribute to the purpose of the concept analysis, for example, articles about neglect in clinical settings such as hospitals or nursing homes. Results from this search provided 11 articles for review.

### Data analysis

The concept of “neglect” within the dementia family caregiving context was examined using the Caron and Bowers^13^ dimensional analysis approach. This method offers a novel avenue to clarify and develop concepts that are relevant to healthcare practice and research. The dimensional analysis illustrates how concepts are social constructs and encompass a variety of contexts and perspectives. By using this method, 1 can discern the relationships between the concept and their social, historical, and cultural influences.^13^ The process of developing a concept analysis in accordance with Caron and Bowers’ method involves 4 basic processes: The first step involves defining neglect as a social concept within the caregiving context. We then examine the concept from multiple perspectives, considering both caregivers and care recipients, to capture diverse experiences and interpretations. The third step is to analyze how these perspectives intersect and influence the concept of neglect. Lastly, we identify underlying assumptions about caregiving, societal norms, and dementia, which shape the understanding of neglect. Through this method, we aim to achieve a thorough and nuanced comprehension of neglect, reflecting its complexity in the specific setting of dementia care.^13^

### Rationale for using dimensional analysis

The current analysis goes beyond a universal definition of neglect as a concept. Dimensional analysis is the best approach for understanding this concept since it needs to be recognized from multiple perspectives as part of different contexts. It is known that neglect events among older adults can vary by culture and context.^30^ Moreover, taking care of a person who has dementia poses unique challenges and burdens for caregivers due to the particularly difficult behavioral symptomatology associated with this disease. For example, Connors et al,^16^ found that care activities involving neuropsychiatric symptoms, function, and general health of care recipients with dementia are strong predictors of burden . There is strong evidence on the relationship between caregiver burden and the use of abusive and neglectful behaviors.^10,11^ Consequently, the use of dimensional analysis fits ideally with the objective of developing an understanding of the meaning and use of the concept of neglect not as a universal phenomenon, but as a particular experience of care, specifically within the context of dementia family caregiving and from the different perspectives of the parties involved.

## Results

### Overview of the concept

The concept of elder neglect, particularly within the context of dementia family caregiving, presents a unique set of challenges that extend beyond the limitations of existing measurement tools. While the lack of standardized instruments for accurately measuring elder neglect is a concern, it is distinct from the conceptual clarity of neglect itself. Indeed, many studies rely on assessments of ‘skipped care’ as an indicator of neglect. However, it is important to recognize that ‘skipped care’—the omission of necessary care tasks—may not fully encapsulate the multifaceted nature of neglect. This is especially true in dementia care, where the dynamics of caregiving are complex and influenced by various factors. Dimensions identified from this concept analysis included (a) expectations of unmet needs (b) maladaptive behavior, and (c) feelings of guilt.

Table 1 presents an overview of the literature on neglect in the context of dementia family caregiving. Out of the 11 articles reviewed, most considered neglect as a subset or category of elder abuse. Five articles provided a conceptualization of neglect specific to their study samples. For instance, Beach and Schulz^31^ noted that caregivers with higher levels of stress and burden tend to forget or fail to meet the more basic needs of their family members living with dementia, which may increase the risk for neglectful events . Following a more operational definition, Fang and colleagues used 6 items from Pillemer and Finkelhor (1988) criteria to document unmet care needs^32^: not providing meals, nutrition, access to health services, personal hygiene, residential/safe environment, safety devices, assistance/assistance with housework.^33-35^ Pickering et al^4^ suggested 3 items that can be used to determine whether a caregiver engaged in negligent behavior toward the person with dementia: unmet needs related to activities of daily living (ADL); lack of oral care, and any other action that subsequently elicited feelings of guilt or embarrassment in the caregiver.

**Table 1.** Articles included in the review, dimensions, and perspectives.

Studies included in review	Study design	Main dimension discussed in the article	Perspectives	
			Caregiver	Older adult living with dementia
• Beach, S. R., & Schulz, R. (2017). Family caregiver factors associated with unmet needs for care of older adults	Quantitative: Correlational secondary data analysis	Expectations of unmet needs	Yes	Yes
• Fang, B., & Yan, E. (2021). Abuse of older persons with cognitive and physical impairments: Comparing percentages across informants and operational definitions	Quantitative: Descriptive secondary data analysis	Expectations of unmet needs	Yes	Yes
• Fang, B., Yan, E., Yang, X., & Pei, Y. (2021). Association between caregiver neurotic personality trait and elder abuse: Investigating the moderating role of change in the level of caregiver perceived burden	Quantitative: Correlational secondary data analysis	Expectations of unmet needs	Yes	Yes
• Tarzian, A. J. (2022). Whose neglect? Exploring patient and caregiver boundaries in advanced dementia	A case study	Expectations of unmet needs	Yes	Yes
• Gallego-Alberto, L., Losada, A., Cabrera, I., Romero-Moreno, R., Pérez-Miguel, A., Pedroso-Chaparro, M. D. S., & Márquez-González, M. (2022). “I feel guilty”. Exploring guilt-related dynamics in family caregivers of people with dementia	Qualitative: Narrative analysis	Feelings of guilt	Yes	No
• Mahomed, A., & Pretorius, C. (2022). Understanding the lived experiences of family caregivers of individuals with dementia in Soweto, a South African Township	Qualitative: Reflective Thematic analysis (RTA)	Feelings of guilt	Yes	No
• Prunty, M. M., & Foli, K. J. (2019). Guilt experienced by caregivers to individuals with dementia: A concept analysis	Concept analysis based on Walker and Avant	Feelings of guilt	Yes	No
• Caputo A. (2021). The emotional experience of caregiving in dementia: Feelings of guilt and ambivalence underlying narratives of family caregivers	Mixed method design	Feelings of guilt	Yes	Yes
• Ali, S., & Bokharey, I. Z. (2016). Caregiving in dementia: Emotional and behavioral challenges	Qualitative: Interpretative phenomenological analysis (IPA)	Maladaptive behavior	Yes	No
• Fang, B., Liu, H., & Yan, E. (2021). Association between caregiver depression and elder mistreatment-examining the moderating effect of care recipient neuropsychiatric symptoms and caregiver-perceived burden	Quantitative: Correlational secondary data analysis	Maladaptive behavior	Yes	Yes
• Pickering, C. E. Z., Yefimova, M., Maxwell, C., Puga, F., & Sullivan, T. (2020). Daily context for abusive and neglectful behavior in family caregiving for dementia	Quantitative: Correlational study using multilevel modeling	Maladaptive behavior	Yes	Yes

**Figure 2.**
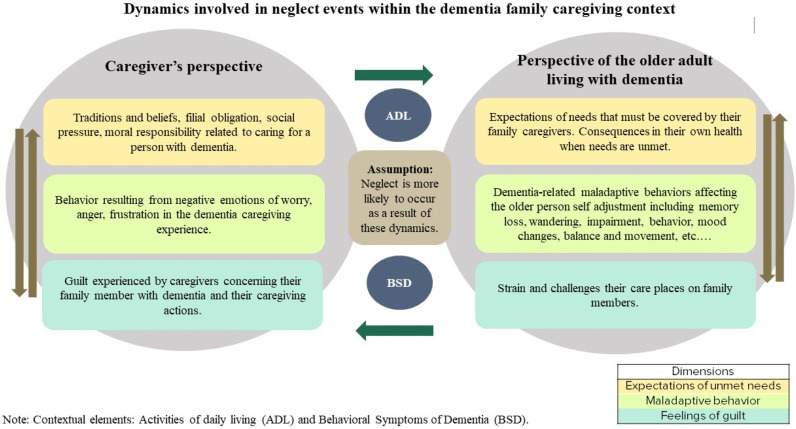
Concept analysis of neglect using Caron and Bowers’ (2000) dimensional analysis method.

The results of Caron and Bowers’s dimensional analysis method,^13^ which explores the relationships between concepts, perspectives, and contexts, are presented in a way that acknowledges these distinctions, as illustrated in Figure 2. The concept of neglect is considered from the perspective of older adults living with dementia and their family caregivers.

### Dimensions, perceptions, and contextual factors

#### Expectations of unmet needs

The first dimension of the concept of neglect in the context of family caregiving for dementia is the expectation of unmet needs, which refers to all tasks that are expected to be fulfilled when taking care of a person with dementia. However, these expectations can vary based on caregivers’ experiences and the clinical conditions of the person with dementia. From the caregivers’ perspective on expectations of unmet needs, the pressure imposed by societal norms and the moral responsibility associated with caring for someone with dementia can significantly impact their well-being.^36-38^ These expectations of unmet needs interact with 2 other dimensions: maladaptive behavior and feelings of guilt. The challenges of dealing with dementia symptoms may lead caregivers to adopt maladaptive behaviors as coping mechanisms, resulting in neglectful behavior and feelings of guilt.

Fang and Yan^33^ offer valuable insights into the traditions and beliefs linked to a filial obligation from the perspective of care recipients living with dementia . Filial obligations encompass a set of deeply rooted traditions and beliefs, emphasizing the expectation that children should fulfill their parents’ needs. These obligations encompass a range of expectations, such as showing profound respect to parents, shouldering caregiving responsibilities, providing financial support, and involving them in important decision-making processes.^38-40^ For individuals living with dementia, their children’s failure to meet these expected needs can be perceived as neglect from the older adult’s viewpoint.

Continuing with the perspective of the older adult living with dementia, when neglect is present within the family caregiving context, we cannot ignore the direct consequences on their health, which may include dehydration, depression, diarrhea, malnutrition, and repetitive medical visits due to possible failure to monitor medical care.^33^

Important contextual elements such as dependence in ADLs were identified as contributing factors to define neglect. For example, Beach and Schulz^31^ found that older adults living with dementia who needed assistance with more than 2 ADLs and those with greater levels of cognitive impairment were more likely to report unmet needs . Additionally, behavioral symptoms of dementia (BSD) are another contextual element within the dynamics of dementia family caregiving. Caregivers experiencing stress related to BSD are more likely to engage in neglectful behavior, which may involve failing to meet the needs of their family members, such as providing assistance with ADLs.^4^

#### Maladaptive behavior

The second dimension of neglect in the context of dementia family caregiving revolves around maladaptive behavior. It is crucial to clarify that individuals exhibiting maladaptive behavior face challenges in adjusting to and participating in specific situations or activities.^41^ Neglect, in itself, can be considered a form of maladaptive behavior stemming from negative emotions like worry, anger, and frustration experienced during the dementia caregiving journey.^36^ BSDs also continue to play a significant role as contextual elements in this aspect. Several studies have highlighted the emergence of neglect as a manifestation of maladaptive behavior among caregivers, especially when coping with the burden of caring for their relatives’ dementia symptoms.^4,31,34,36,42^

Caregivers with higher levels of negative affect (anxiety, depression, stress, sadness, worry, guilt, shame, anger, and envy) and exaggerated reactivity (excessive or heightened response to a stimulus or situation) to caregiving stressors were found to be more likely to engage in neglectful behaviors.^35^ Similarly, other 2 studies revealed that caregivers experiencing elevated burden, stress, and negative psychosocial well-being were also prone to resorting to neglectful behaviors towards their relatives with dementia.^31,43^

While most articles focused on the caregiver perspective, 1 noteworthy case study provided insight into the viewpoint of an older person living with dementia. This study described the reaction of a father living with dementia when his daughter who experienced burden and stress wanted outside help to decrease her father’s level of unmet needs/neglect: “He often became abrasive and angered when anyone tried to help bathe him, turn him in bed, or spoon feed him”.^44^ It is clear that BSDs, which can also be seen as a form of maladaptive behavior among older individuals with dementia, play an important role and are related to the manifestation of maladaptive behaviors exhibited by caregivers when they use neglectful behaviors in response to caregiving stress.

#### Feelings of guilt

The third dimension identified in the context of neglect within dementia family caregiving is the feeling of guilt. This dimension interacts with the expectations of unmet needs, and maladaptive behaviors. Prunty and Foli^37^ mentioned that in contexts where caregivers believe they have a moral obligation to care for their relatives with dementia but have broken that moral standard through their maladaptive behavior or thoughts, guilt arises . Furthermore, different sources of guilt were described within the dementia family caregiving context,^45^ including guilt for experiencing negative emotions towards their relative; guilt triggered by changes in the relationship with the person living with dementia; guilt resulting from the use of neglectful behaviors towards themselves and their relatives; guilt induced by their relative’s perception of unmet needs; and even guilt imposed by third parties, who may unjustly label them as criminal and perpetrators of neglect.^42^

Guilt is a significant indicator, revealing that caregivers are aware of their participation in neglectful behaviors.^38^ This aspect is crucial, as neglect is often assessed in a context-insensitive way, simply as an omission of care. Yet, the existence of guilt suggests an element of intentionality, which resonates more with the established definitions of elder abuse. Caregivers’ acknowledgment of guilt underscores their understanding of the importance and necessity of care activities, providing deeper insights into the complexities of the caregiving experience.

The experience of guilt in dementia caregiving extends beyond caregivers to include the perspective of older adults living with dementia. These individuals often play a significant role in the dynamics of guilt, particularly in light of the strong moral devotion and responsibility felt towards in-home care, a sentiment deeply rooted in family ties.^38^ Their awareness of the strain and challenges their care places on family members can contribute to feelings of guilt, highlighting the complex emotional interplay in the caregiver/care-recipient relationship, where both parties are influenced by a sense of duty and affection shaped by familial bonds.

Acknowledging and understanding the role of guilt provides a profound glimpse into the emotional intricacies that define the caregiving experience, emphasizing the need for compassionate and comprehensive support for both the caregiver and the older adult living with dementia.

## Discussion

The findings of this concept analysis reveal the complex nature of neglect within dementia family caregiving, highlighting its multidimensionality. The study underscores the importance of considering neglect as a distinct and context-dependent experience, which can vary based on the perspectives of both the caregiver and the recipient. Neglect in the dementia caregiving context can be defined as the consequence or result of a complex interaction between expectations of unmet needs, maladaptive behaviors, and feelings of guilt experienced by both the caregiver and the relative living with dementia. Moreover, within this intricate context, neglect can be influenced by certain contextual elements, including activities of daily living, behavioral symptoms of the relative living with dementia, and basic self-care daily living skills. These factors may act as moderators in shaping the outcome of neglect. This concept analysis highlights the importance of understanding neglect as a social construct influenced by cultural, emotional, and moral factors. This recognition is crucial for developing effective interventions and support systems that address the unique challenges faced by caregivers in the context of dementia.

The analysis highlights the need for measurement tools that capture the complexity of neglect in caregiving. Current methods often overlook the multifaceted nature of neglect, leading to oversimplification and potential misinterpretation. This study’s comprehensive definition of neglect emphasizes the importance of tools that reflect its various dimensions and underlying causes. A critical evaluation of existing tools against this broader understanding can reveal deficiencies and opportunities for enhancement. The study suggests refining or restructuring existing measurement instruments to represent the nuanced reality of neglect more accurately in caregiver relationships, aligning them with the insights gained from this analysis.

Recognizing neglect as a dyadic phenomenon emphasizes the significance of interactions between caregivers and individuals with dementia in shaping neglect outcomes. To account for the intricacies of caregiving relationships, it is imperative to develop context-specific measurement tools. These tools should consider the complex dynamics and nuances that exist within caregiving settings, enabling a more accurate and comprehensive assessment of neglect. Additionally, it is worth noting that the lack of standardized definitions and criteria for neglect has been supported by previous literature, as evidenced by Dong.^27^ This highlights the urgency for researchers to address this gap by devising contextually appropriate and consistent measures that align with a comprehensive understanding of neglect in caregiving contexts.

Research on interventions to prevent or mitigate elder abuse, especially in the context of family caregiving for people with dementia in the community, remains limited. Daly and Butcher^46^ reported interventions such as education and training programs for caregivers, multidisciplinary teams, and screening tools, there is insufficient evidence supporting their effectiveness in addressing neglect . Identifying the most effective approaches is challenging due to the lack of clear evidence. Potential unexplored intervention targets could be investigated through prospective studies to address neglect in the context of dementia family caregiving. These targets include comprehensive caregiver training programs, respite care services to reduce caregiver burden, collaborative care models with multidisciplinary teams, technology-based interventions like remote monitoring systems and caregiver support apps, and public awareness campaigns to increase knowledge and understanding of neglect in dementia caregiving. Prospective studies on these interventions would contribute to evidence-based practices and better support for caregivers and individuals with dementia.

The relational dimension of neglect is important from the perspective of both the caregiver and the person living with dementia. Future research should focus on investigating and refining measures to better assess the relational aspects of neglect in caregiving relationships. By considering neglect as a dyadic phenomenon, researchers can delve into the intricate interactions and exchanges of care, communication, and emotions between the caregiver and the person living with dementia. By doing so, we can enhance our ability to prevent, detect, and intervene effectively in cases of elder neglect. This knowledge becomes instrumental in advocating for the well-being of both caregivers and vulnerable older adults, fostering a society that truly values and safeguards the dignity and rights of all its members.

### Limitations

This concept analysis has certain limitations. The search for relevant literature was constrained by the lack of studies exclusively focusing on neglect, as most studies integrate neglect with abuse, making it challenging to distinguish and understand neglect as a unique phenomenon. Additionally, a significant limitation in the existing body of research is the lack of cultural and contextual detail. Many studies do not sufficiently account for the diverse cultural, social, and environmental factors that can influence the manifestation and perception of neglect, especially in the context of dementia family caregiving. This omission can lead to an incomplete understanding of how neglect varies across different cultural and socio-economic backgrounds.

To enrich and refine this concept analysis, it is imperative to foster a more nuanced view of neglect, considering it as a unique and independent variable, distinct from abuse. Future research should aim to fill these gaps by incorporating cultural and contextual nuances, which would provide a more comprehensive and accurate picture of neglect in diverse caregiving settings.

Despite these limitations, the concept analysis offers valuable insights into the multifaceted nature of neglect within dementia family caregiving. It emphasizes the need for further research to comprehensively understand and address neglect in this context. Future studies can build upon the dimensions identified in this analysis and explore the effectiveness of interventions to prevent neglect, support caregivers, and promote the well-being of individuals living with dementia. Additionally, researchers should continue to advocate for clearer definitions and standardized measurement tools to advance the understanding of neglect in dementia family caregiving and elder abuse research.

### Implications for practice

Primary care physicians, mental health professionals, and other health professionals, especially nurses, play a crucial role in the prevention of elder abuse and neglect, particularly in the context of dementia caregiving.^47^ The active participation of multidisciplinary teams is vital for understanding and addressing neglect effectively. This study provides an important initial overview of neglect in this specific caregiving context. Health professionals can further contribute by educating family caregivers about the risk factors that may lead to the use of neglectful behaviors, emphasizing a non-judgmental approach that avoids criminalizing or shaming situations. They can also provide valuable information on available resources and support services to prevent and address neglect. However, to facilitate these practices healthcare professionals require frameworks and a solid foundation to guide their prevention efforts effectively.

## Conclusion

Healthcare research in primary care, nursing, and other healthcare settings can significantly contribute to the effort to prevent negative care outcomes, including neglect situations among family caregivers with dementia. Understanding the concept of neglect in different contexts and perspectives is essential to drive interventions and policy changes that recognize this phenomenon and support both older adults living with dementia and their caregivers. Since neglect only occurs in the context of caregiving, primary care physicians, nurses, and mental health professionals are poised to lead the field in addressing neglect and supporting the provision of high-quality care in family caregiving. This concept analysis considered neglect phenomena from the perspective of older adults living with dementia and their family caregivers. Dimensions identified from this concept analysis included (a) unmet need expectations (b) maladaptive behavior and (c) feelings of guilt which can be important intervention targets to prevent elder neglect in the dementia family caregiving context. Moving forward, future research should further explore neglect phenomena in diverse caregiving settings to advance the prevention of elder neglect. By continuing to investigate and address neglect, nursing research can make substantial contributions toward improving the well-being and care outcomes of individuals with dementia and their caregivers.
